# Challenges With the Intolerance of Biologics in Severe Eosinophilic Asthma: The Risk of Exacerbations Is Time-Dependent With the Rise of Eosinophils

**DOI:** 10.7759/cureus.74523

**Published:** 2024-11-26

**Authors:** Arturo Cortes-Telles, Yuri Noemí Pou-Aguilar, Esperanza Figueroa-Hurtado, Saul Vazquez-Lopez, Diana L Ortiz

**Affiliations:** 1 Respiratory Diseases Clinic, Hospital Regional de Alta Especialidad de la Península de Yucatan, Merida, MEX

**Keywords:** biologics, blood eosinophils, exacerbations, intolerance, uncontrolled asthma

## Abstract

Patients with severe eosinophilic asthma (SEA) can benefit from biologic therapy but some subjects may present an immune-mediated side effect. These patients will not meet the treatment goals and might have an increased risk of exacerbations. Monitoring these patients by determining blood eosinophil (BE) levels could be one of the tools that may allow a follow-up to prevent a worsening of asthma or exacerbations.

The patient was a 50-year-old female with a diagnosis of SEA who had six asthma exacerbations in the last year. Baseline spirometry showed a forced expiratory volume in one second (FEV1) (L) of 0.80 with 34%p (z-score: -4.18) post bronchodilator (BD) and a BE count of 15% (1080 µL cells). After three doses of benralizumab, the spirometry showed an FEV1 (L) of 2.31 with 97%p (z-score: -0.21) post BD and a BE count of 0%. No exacerbations were reported while the patient received biologic medication, but it had to be stopped due to a dermatological allergic reaction. The patient was well-controlled for four months after stopping biologic medication and the BE count also increased, associated with two emergency visits due to exacerbations despite maintenance therapy.

Although intolerance or hypersensitivity reactions to the treatment with biologics in asthmatic patients are rare, these patients must discontinue treatment, which increases their risk of exacerbations. In routine medical clinical practice, an easy approach can be made with the follow-up of an accessible and inexpensive test, i.e., the BE count. Its increments should trigger an alert for a closer follow-up and early therapy adjustments.

## Introduction

Although biologic medications have shown an acceptable safety and efficacy profile in the treatment of severe eosinophilic asthma (SAE), there is a need to monitor those patients who discontinue the medication after experiencing a safety event. This is challenging due to limited information, a lack of standardized criteria, and parameters to be measured [1]. Blood eosinophil (BE) levels have become the most robust biomarker, thus allowing monitoring and prevention of subsequent risk of asthma worsening.

Type-2 inflammatory phenotype (Th2) occurs in approximately 50% of patients with severe asthma [2]. Th2 inflammation is triggered when the organism recognizes allergens. Dendritic cells, which are part of the innate system, shift the T-lymphocyte differentiation into Th2, initiating and sustaining allergic inflammation associated with asthma [3]. Th2 and innate lymphoid cells, as part of the adaptive immune system, increment the levels of cytokines IL-4, IL-5, and IL-13 that generate and regulate inflammation [1]. A high Th2 endotype is frequently recognized by blood or sputum eosinophilia, an increased exhaled fraction of nitric oxide (FeNO), and may be accompanied by atopy [2].

Patients with SEA who are not properly controlled with therapy based on high-dose inhaled corticosteroids (ICS) plus long-acting β-agonists (LABA) are candidates to receive anti-interleukin-5 receptor (anti-IL-5R) agents as added therapy [4]. Among anti-IL-5R agents, benralizumab reduced the annual rate of exacerbations and hospitalization/emergency room access by up to 70% and 93%, respectively, and induced a significant reduction in oral corticosteroids (OCS) use and even 52% of the patients stopped the use of OCS [4-6]. Although benralizumab has an acceptable safety profile, around 2.1-4.0% [5,7] of patients stop the medication, and side effects are among the main reasons. More frequent adverse effects are headache (8%), pharyngitis (4%), cough (3%), and hypersensitivity reactions represent only 1% of reported cases [5,7]. Once the biologic treatment is stopped by a safety event, there is a high risk of exacerbations and an increment in BE levels [8] but the precise time of eosinophils retrieval has been poorly described.

## Case presentation

The patient was a 50-year-old female who never smoked and had a familiar history of asthma, biomass exposure (stopped by the age of 20), and allergic rhinosinusitis. She was referred to our respiratory disease clinic with a diagnosis of uncontrolled asthma despite mild-to-moderate ICS + LABA combination with six exacerbations in the past year using several courses of OCS (five days of 50 mg of prednisone) on each exacerbation.

**Figure 1:**
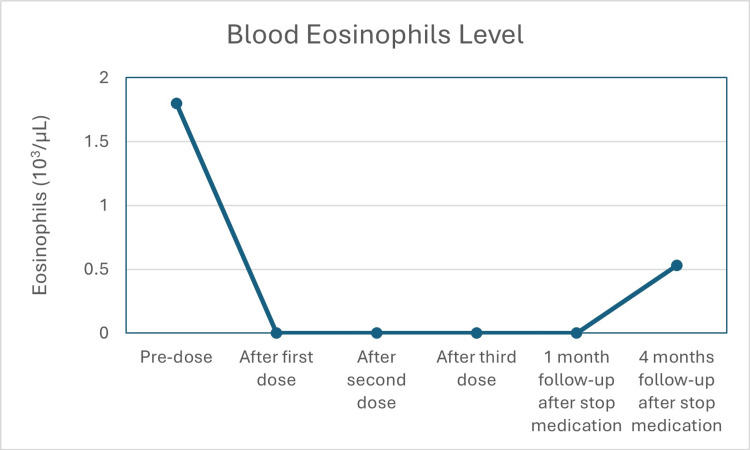
Laboratory tests.

A detailed clinical history revealed that the main triggers of exacerbations were weather changes, viral infections, and dust mite exposure. Her baseline spirometry showed a severe obstructive pattern with a post-bronchodilator (post-BD) forced expiratory volume in one second (FEV1) (L) of 0.80 with 34%p (z-score: -4.18). There was a 21% reversibility. The baseline BE count was 15% (1080 µL cells) (Table 1 and Figure 1).

**Table 1:** Pulmonary function tests (baseline/follow-up). % Pred = percentage of predicted value; % Change = percentage of change; FVC = forced vital capacity; FEV1 = forced expiratory volume in one second; FEV1/FVC = forced expiratory volume in one second/forced vital capacity. (a) The z-score is the result of combining the predicted percentage and the variability between subjects. This number represents the variability of lung function related to age and height. The spirometry value is compared with that of healthy subjects. Current standards of the American Thoracic Society and European Respiratory Society interpretation suggest that spirometry values are reported as z-score, as well as the predicted value.

		FVC (L)	FEV1 (L)	FEV1/FVC	Best FEV1/FVC
Pre-dose					
Pre-bronchodilator	% Pred	57.00	28.00		0.38
	Z-score	-2.73	-4.55	-6.22	
Post-bronchodilator	% Pred	78.00	34.00		0.34
	Z-score	-1.43	-4.18	-8.84	
	% Change	36.00	21.00	-11	
After the first dose					
Pre-bronchodilator	% Pred	101	74.00		0.79
	Z-score	0.06	-1.61	-3.17	
Post-bronchodilator	% Pred	104	73.00		0.55
	Z-score	0.28	-1.73	-3.68	
	% Change	3.00	-2.00	-6.00	
After the second dose					
Pre-bronchodilator	% Pred	108	83.00		0.79
	Z-score	0.53	-1.05	-2.79	
Post-bronchodilator	% Pred	105	76.00		0.56
	Z-score	0.33	-1.54	-3.40	
	% Change	-3.00	-9.00	-7.00	
After the third dose					
Pre-bronchodilator	% Pred	118	95.00		0.79
	Z-score	1.15	-0.33	-2.39	
Post-bronchodilator	% Pred	119	97.00		0.64
	Z-score	1.21	-0.21	-2.26	
	% Change	1.00	2.00	1.00	

According to the findings, the patient exhibited a Th2 phenotype and, therefore, was suitable for biologics therapy, and the class of anti-IL-5/anti-IL-5R was considered the best option together with up-titration with a single-inhaler triple-therapy based on beclomethasone/formoterol/glycopyrronium 100/6/12.5 mcg, two inhalations every 12 hours. In addition, she was placed under treatment for allergic rhinosinusitis with nasal budesonide 64 mcg twice per day (BID) on each nostril, levocetirizine 5 mg once a day (QD), and ocular olopatadine 0.2% as needed.

After three doses of benralizumab, her spirometry showed a persistent obstructive pattern (FEV1/forced vital capacity = 0.70 pre-BD) but an FEV1 between normal ranges (FEV1 (L) = 2.26 with 95%p, z-score: -0.33) pre-BD and a negative bronchodilator response. The BE eosinophils count was 0% (Table 1 and Figure 1).

The Asthma Control Test (ACT) showed asthma control while she maintained good treatment adherence (Table 2).

**Table 2:** The Asthma Control Test (ACT) and Test of Adherence to Inhalers (TAI) scores.

	2 March 2023	18 May 2023	6 June 2023	8 August 2023
	Pre-dose	After the first dose	After the second dose	After the third dose
ACT total score	6	23	25	25
TAI	53	54	54	54

No exacerbations were reported while the patient received anti-IL-5/anti-IL-5R medication without subsequent need for OCS and it was possible to de-escalate inhaled triple therapy to moderate ICS + LABA combination (budesonide/formoterol 160/4.5 mcg, BID).

After the third dose, the patient reported a dermatological allergic reaction characterized by itching all over the body altogether with erythema and swelling of the periorbicular area, lips, and neck corresponding to grade 2 (10-30% of body surface area). She was placed on OCS (prednisone 50 mg QD), antihistamines (loratadine 10 mg QD) as well as antileukotrienes (montelukast 10 mg QD) for five days and anti-IL-5/anti-IL-5R was stopped thereafter. She was re-evaluated one week after the reaction with a good response to treatment.

Four months after stopping biologic medication, the patient had multiple exacerbations despite maintenance with moderate ICS + LABA combination therapy. Two exacerbations needed evaluation in the emergency department and courses of OCS 50 mg once a day for six days (300 mg total per event) were prescribed. BE levels showed increments from 0% to 7% (530 µL cells).

## Discussion

The use of treatments with biological molecules has been progressively increasing due to their healing capacity. Patients with SAE have shown good therapeutic responses with antieosinophilic treatments [9,10]. However, its mechanisms of action are not without risks. Adverse reactions associated with immunomodulators can have significant clinical repercussions [11]. Hypersensitivity reactions have been reported with low rates of 1-3.2% [12] for all targeting IL-5 drugs and also exhibit overlapping safety profiles that make the possibility of switching them difficult.

Our clinical case showed that patients with a Th2 phenotype who had an excellent response to this type of anti-IL-5R agent such as benralizumab could face hypersensitive reactions that demand stopping the medication, in this instance, a skin rash that resolved with standard therapy. Four months after stopping treatment with benralizumab, asthma exacerbations occurred twice, and these required the administration of OCS for its control, as she did not respond to inhaled triple therapies. OCS use has been associated with key and frequent adverse reactions such as osteoporosis, osteoporotic fractures, type 2 diabetes, increment of weight, ocular problems such as cataracts, and blood hypertension. All these conditions impact the health status and quality of life of the patients and increase the medical costs [13].

In addition, we found an increment in the BE level associated with asthma exacerbation. Elevated eosinophil levels have been used as a biomarker associated with SEA. The increase in eosinophils has been measured in both sputum and blood. Although eosinophilia in sputum was initially considered to be the reliable biomarker, more recently it has been validated that elevated BE levels also have a positive association with SAE and with eosinophil levels measured in sputum [14].

High BE levels have been linked to higher rates of severe exacerbations, indicating less disease control. Nayyar et al. [15] reported that 64.4% of asthmatic patients had at least one BE level ⩾300 µL cells in the previous 12 months of their hospital admission. A post hoc analysis with mepolizumab reported increases in BE level and loss of asthma control from 12 weeks after stopping treatment [8]. Mallah et al. [16] found that high BE levels (⩾200 µL cells) are associated with asthma exacerbations (OR: 1.31), visits to the clinics (OR: 1.46), and emergency department visits (OR: 1.63). Hirano et al. [17] reported that BE levels could also be associated with the status of lung function, all-cause mortality, and response to biological therapies. All these findings support the idea that BE levels could have a predictive value, as shown in our case.

So, in clinical practice, the BE levels could be used to monitor asthma exacerbations, as its increments predict a higher risk of occurrence. At present, the use of BE levels to predict the worsening of asthma presents challenges regarding their optimal use in which levels are associated with higher frequency of exacerbations and how BE levels can influence the therapy for patients with SAE.

## Conclusions

The management of asthma includes several targets: control of asthma symptoms, decrease the number of asthma exacerbations, and protect lung function. Anti-IL-5/anti-IL-5R has proven to be a very effective added therapeutic option for asthma patients to reach these goals. However, its potential to cause hypersensitivity or safety reactions that force suspend the treatment increases the risk of asthma exacerbations.

As the time to suffer a new asthma exacerbation is variable, as in our case, it lasted four months, a practical approach can be used with the follow-up of an accessible and inexpensive test, i.e., the blood eosinophil count. Its increments should trigger an alert for a closer follow-up and early therapy adjustments.
